# GAP43, a novel metastasis promoter in non-small cell lung cancer

**DOI:** 10.1186/s12967-018-1682-5

**Published:** 2018-11-12

**Authors:** Fanrong Zhang, Lisha Ying, Jiaoyue Jin, Jianguo Feng, Kaiyan Chen, Minran Huang, Yingxue Wu, Herbert Yu, Dan Su

**Affiliations:** 1 Department of Breast Surgery, Zhejiang Cancer Hospital, Hangzhou, China; 2 Cancer Research Institute, Zhejiang Cancer Hospital & Key Laboratory Diagnosis and Treatment Technology on Thoracic Oncology of Zhejiang Province, No. 1 East Banshan Road, Hangzhou, 310022 China; 3 Department of Pathology, Zhejiang Cancer Hospital, Hangzhou, China; 4 Cancer Epidemiology Program, University of Hawaii Cancer Center, Honolulu, HI USA

**Keywords:** Non-small cell lung cancer, Metastasis, High-throughput assays, GAP43, F-actin

## Abstract

**Background:**

Brain metastasis is an extremely serious sequela with a dismal prognosis in non-small cell lung cancer (NSCLC). The present study aimed to identify novel biomarkers and potential therapeutic targets for brain metastases of NSCLC.

**Methods:**

We performed high-throughput Luminex assays to profile the transcriptional levels of 36 genes in 70 operable NSCLC patients, among whom 37 developed brain metastases as the first relapse within 3 years after surgery. The Cox proportional hazards regression model was used to evaluate the association between genes and brain metastases. Wound healing assay and transwell assay was carried out to estimate the function of target gene in vitro. And left ventricular injection on nude mice was used to evaluate the effect of target gene in vivo.

**Results:**

Growth-associated protein 43 (GAP43) was found to be related to brain metastasis. Multivariate Cox regression analysis showed that NSCLC patients with elevated GAP43 had a 3.29-fold increase in the risk for brain metastasis compared with those with low levels (95% confidence interval: 1.55–7.00; *P* = 0.002). Kaplan–Meier survival curves revealed that GAP43 was also associated with overall survival. Analysis of a cohort of 1926 NSCLC patients showed similar results: patients with high levels of GAP43 had worse progression-free and overall survival rates. Furthermore, in vitro experiments showed that GAP43 facilitated cell migration. Animal studies demonstrated that GAP43-silenced NSCLC cells were less likely to metastasize to the brain and bone than control cells. Immunofluorescence and F-actin/G-actin in vivo assays indicated that GAP43 knockdown triggered depolymerization of the F-actin cytoskeleton. Rho GTPase activation assays showed that Rac1 was deactivated after GAP43 was silenced.

**Conclusions:**

Our findings suggest that GAP43 is an independent predictor of NSCLC brain metastasis and that it may facilitate metastasis by regulating the Rac1/F-actin pathway.

## Background

Brain metastases are one of the most severe late sequelae of malignancies. Approximately 20–30% of patients with non-small cell lung cancer (NSCLC) develop brain metastases during the course of disease, and 10–20% of patients present with a synchronous diagnosis of brain metastases and a primary tumor [1, 2]. Due to the enhanced control of extracranial disease and wide availability of magnetic resonance imaging, an increasing proportion of NSCLC patients with brain metastases is expected in the coming years [3–5]. The treatment and outcomes of brain metastasis remain unsatisfactory, although presently, appropriate targeted therapies can be offered to some NSCLC patients with EGFR mutation or ALK rearrangement [1, 6, 7]. Therefore, exploration of the underlying mechanisms to identify potential therapeutic targets is valuable.

Recently, a growing number of studies have focused on organ-specific metastasis. Notably, a successful brain metastatic cell requires specialized mechanisms to traverse the blood–brain barrier (BBB), a selective barrier formed by cerebrovascular endothelial cells that exerts the foremost control over the brain microenvironment [8, 9]. As reported, tumor cells arrested at the brain vascular branch can adhere to the endothelium and secrete certain cytokines, such as matrix metalloproteinases (MMPs), placental growth factor (PLGF) and vascular endothelial growth factor (VEGF), to break the tight junction and promote retraction of the endothelial monolayer [10–12]. Accompanied by the rearrangements of actin fibers regulated by Rho GTPases, cancer cells can protrude pseudopodia and extravasate in a manner similar to leukocytes [13, 14]. The infiltrated cancer cells must then co-opt cerebral microvessels and escape from being killed by reactive astrocytes [15, 16]. Nonetheless, much is still unknown about the exact mechanisms of brain metastasis, and little has been translated to clinical research and application.

Using a high-throughput Luminex assay of NSCLC tissues, the present study identified growth associated protein 43 (GAP43), an axonal membrane protein, as an independent predictor of brain metastasis. Both in vivo and in vitro experiments indicated that GAP43 can facilitate tumor metastasis. Further study of the mechanisms showed that GAP43 might promote cell migration by activating Rac1 and mediating F-actin cytoskeleton polymerization.

## Methods

### Patients and tissues

In total, 70 patients with operable NSCLC were collected from our previously constructed database [17]. Among them, 37 patients developed brain metastases as the first relapse event within 3 years after surgery, and 5 patients underwent brain metastatic tumor resection. Their formalin-fixed and paraffin-embedded (FFPE) tissues were obtained from the Tissue Bank of Zhejiang Cancer Hospital. The follow-up proceeded as previously described [17]. The median follow-up time was 42 (range 5–108) months. During follow-up, 2 of 33 3-year recurrence-free patients relapsed, 30 of 70 patients died of tumor progression, and 1 of 70 patients was lost to follow-up. All patients provided informed consent before surgery, and the study was approved by the Institutional Review Committee of Zhejiang Cancer Hospital.

### Luminex assay

Based on previous publications [18–20], we selected 36 genes hypothesized to be associated with brain metastasis. These genes represented three functional categories: brain growth and metabolism, epithelial–mesenchymal transition and cytokines. Six 10-μm-thick paraffin sections were cut from each NSCLC FFPE sample for the Luminex assay. Tissue homogenates were prepared using a QuantiGene Sample Processing Kit for FFPE Tissues (Affymetrix, Santa Clara, CA, USA) according to the manufacturer’s protocol. A QuantiGene plex 2.0 Reagent System (Affymetrix) was used to capture target RNA and amplify the signal according to the manufacturer’s recommended procedure. Finally, the signal was detected using a Luminex 200 System (Luminex, Austin, TX, USA).

### Immunohistochemistry (IHC)

Four-micrometre-thick paraffin sections were cut, mounted on slides, deparaffinized in xylene, rehydrated in decreasing ethanol dilutions and incubated in 3% hydrogen peroxide buffer for 30 min. After blocking of endogenous peroxidase activity, the slides were boiled with citrate buffer (pH 6.0) in a pressure cooker for 90 s, followed by blocking of non-specific binding sites with 5% blocking serum for 30 min at room temperature. The sections were then incubated with rabbit anti-GAP43 antibody (Huabio, Hangzhou, China) at a dilution of 1:1200 overnight at 4 °C. Afterwards, 1:2000 diluted goat anti-rabbit secondary antibody (Dako, Glostrup, Denmark) was applied for 30 min. The reaction products were visualized using 3,3′-diaminobenzidine (DAB; Dako), and the sections were lightly counterstained with hematoxylin and mounted. The results were evaluated by two experienced pathologists and photographed.

### Cell culture

The human NSCLC cell lines SK-MES-1, NCI-H1650, NCI-H1975, NCI-H2122, A549, NCI-H838, NCI-H460 and NCI-H661 were purchased from American Type Culture Collection (Manassas, VA, USA). All cells were authenticated by short tandem repeat (STR) profiling and were found to be negative for mycoplasma. SK-MES-1 cells were maintained in Eagle’s minimum essential medium (EMEM; Genom, Hangzhou, China) containing 10% fetal bovine serum (FBS, Gibco); all other cell lines were maintained in RPMI 1640 medium (Gibco) containing 10% FBS. All cells were cultured at 37 °C in a 5% CO_2_ incubator.

### Western blotting

Adherent cells were collected by gentle scraping, washed three times in phosphate-buffered saline (PBS), and lysed in Nonidet P 40 cell lysis buffer supplemented with phosphatase inhibitor cocktail (Cwbio, Beijing, China) and protease inhibitor cocktail (Roche, Mannheim, Germany). A Bradford calorimetric assay (Cwbio) was used to measure the protein concentration of the cell lysates. Thirty micrograms of total protein was separated on a 10% SDS–polyacrylamide gel electrophoresis (SDS-PAGE) gel and then transferred to a polyvinylidene fluoride membrane (Millipore, Bedford, MA, USA). Each membrane was blocked in TBS-Tween-20 (TBS-T) containing 5% non-fat milk at room temperature for 2 h, followed by incubation with anti-GAP43 antibody (1:1000, Huabio, Hangzhou, China) at 4 °C overnight. The next day, the membrane was incubated with an HRP-conjugated secondary antibody (Servicebio, Wuhan, China) at room temperature for 2 h, and the results were detected using an enhanced chemiluminescence (ECL) reagent (Cwbio). Tubulin (1:1000; Antibody Revolution, San Diego, CA, USA) was used as the loading control.

### Reverse transcription-quantitative polymerase chain reaction (RT-qPCR)

Total RNA was extracted from cells using the TRIzol method. The RNA concentration was measured using a microvolume spectrophotometer, and 500 nanograms of RNA was reverse transcribed using a PrimeScript™ RT Reagent Kit (Takara, Dalian, China). Quantitative PCR (qPCR) was carried out as previously described [21]. The primer sequences were as follows: TTCTTGGTGTTGTTATGGCAAG (GAP43 forward), GAGGAAAGTGGACTCCCACAG (GAP43 reverse), GAAGGTGAAGGTCGGAGTC (GAPDH forward), and GAAGATGGTGATGGGATTTC (GAPDH reverse). The expression levels of GAP43 were analyzed using the 2(−Delta Delta C(T)) method [22] and adjusted according to the expression levels of the housekeeping gene GAPDH.

### GAP43 knockdown and overexpression

For transient knockdown, 4 specific siRNAs targeting GAP43 and a negative control siRNA were designed and inserted into a pRNAT-U6.1/Neo vector. For transient overexpression, GAP43 cDNA was subcloned into a p3 × FLAG-CMV-10 vector, and an empty p3 × FLAG-CMV-10 vector was used as the control. Lipofectamine 3000 Reagent (Invitrogen, Carlsbad, CA, USA) was used to carry out the transfections, and after 48 h, the cells were harvested to validate the alterations in GAP43 levels or for subsequent experiments.

For stable GAP43 knockdown, a lentiviral vector was applied. Briefly, the GAP43 knockdown lentiviral vector pHBLV-CMV-shGAP43-3flag-EF1-Luc-T2A-Puro and a negative control lentiviral vector were purchased from Hanbio Biotechnology Co., Ltd., Shanghai, China. 293T cells were co-transfected with lentiviral vectors and the helper plasmids pSPAX2 and pMD2G. After 48 h of transfection, the supernatant was collected and purified by centrifugation followed by ultracentrifugation. NCI-H661 cells at 50% confluence were infected with GAP43 knockdown or negative control lentiviral constructs in 8 µg/ml polybrene. Stably transfected cells were selected using 1 µg/ml puromycin for 2 weeks and maintained in 0.5 µg/ml puromycin. GAP43 knockdown was confirmed by western blotting.

### Wound healing assay

The wound healing assay was conducted using a 2-Well Culture-Insert (Ibidi, Munich, Germany). Cells were seeded into the wells, and after removal of the insert, a 500-μm cell-free gap was formed. Multiple time-lapse images of the gap were taken at a magnification of 100×.

### Transwell migration and invasion assays

Twenty-four-well, 8-μm pore size transwell inserts (Corning, Oneonta, NY, USA) were used. For invasion assays, the inserts were coated with Matrigel (BD, San Jose, CA, USA) before the cells were added. A total of 500 μl of complete medium was added to the lower chamber, and 200 μl of cell suspension (3.0 × 10^4^ NCI-H661 cells for migration, 2.5 × 10^4^ NCI-H661 cells for invasion, 2.0 × 10^4^ NCI-H1650 cells for migration and invasion) in RPMI 1640 supplemented with 1% FBS was added to the upper chamber. After incubation for 24 h, the cells in the upper chamber were removed by gentle wiping using cotton buds, and the cells on the underside of the inserts were fixed with methanol, stained with 0.1% crystal violet, photographed and counted.

### In vivo metastasis assay

BALB/c (nu/nu) male nude mice 4–6 weeks of age purchased from Shanghai Slac Laboratory Animal Co. Ltd. were used, each group with 10 mice. Two hundred microliters of PBS containing 1.0 × 10^6^ cells was injected into the left ventricle of each mouse. Metastatic lesions were monitored every week using bioluminescence imaging (BLI). Briefly, the mice were anaesthetized and injected intraperitoneally with 150 µg of d-luciferin (Yeasen, Shanghai, China) per gram of weight. 10 min later, the bioluminescence was imaged using an IVIS imaging system (Caliper Life Sciences, Alameda, CA, USA) and analyzed using Living Image Software 4.3.1.

### Immunofluorescence

Adherent cells at 80% confluence on an 8-well glass slide (Millipore) were fixed in 4% paraformaldehyde for 15 min and permeabilized with 0.1% Triton X-100 for 5 min. Next, 100 µl of phalloidin conjugate working solution (Abcam, Cambridge, MA, USA) was added to each well of the slide and incubated with the cells for 60 min at room temperature away from light. The cell nuclei were stained with DAPI (Biosharp, Hefei, China) for 10 min at room temperature away from light, and fluoroshield mounting medium (Abcam) was used to seal the slide. Fluorescence was photographed using a confocal microscope.

### F-actin/G-actin ratio in vivo assay

Proteins were extracted and analyzed using an F-actin/G-actin in vivo assay kit (BK037; Cytoskeleton, Denver, CO, USA) based on the manufacturer’s instructions. Briefly, cells were lysed in a detergent-based buffer that dissolved G-actin but not F-actin. A centrifugation step pelleted F-actin while leaving G-actin in the supernatant. Actin in the pellet and supernatant was quantitated by western blotting. The grayscale value of each band was measured using ImageJ software.

### Rac1 activation assay

A Rac1 activation assay kit (STA-405; Cell Biolabs, San Diego, CA, USA) was applied to prepare protein according to the manufacturer’s instructions. Briefly, protein was extracted from cells and divided into two equal parts. One part was used to quantitate total Rac1. The other part was incubated with the p21-binding domain (PBD) of p21-activated protein kinase (PAK) Agarose beads to specifically bind and pull-down active Rac1. Next, total Rac1 and active Rac1 were detected by western blotting.

### Statistical analysis

The expression index of each gene from the Luminex assay was averaged in two groups according to the median and then analyzed as categorical variables. The Cox proportional hazards regression model was applied to evaluate the brain metastasis-prognostic value of each gene. The Kaplan–Meier estimator was used to generate brain metastasis-free survival curves, and a log-rank test was used to determine significant differences. A web application called KM plotter (http://kmplot.com/analysis/index.php?p=service&cancer=lung) was employed to obtain the progression-free survival and overall survival curves in public database of lung cancer. Correlations between GAP43 expression and clinicopathological factors were examined using a Chi square test and Mann–Whitney U test. Every experiment was repeated at least 3 times. The results of qPCR, western blotting and cell migration and invasion assays were analyzed using Student’s t-test, and the BLI results were analyzed with a Mann–Whitney U test. IBM SPSS Statistics 24.0 software was used for data processing. A *P* value < 0.05 was considered statistically significant.

## Results

### Patient characteristics

Thirty-seven NSCLC patients who developed brain metastases as the first relapse within 3 years after surgery were selected. Thirty-three patients were successfully matched with 3-year recurrence-free patients according to age, sex, smoking history, histology, grade and clinical stage. Their clinicopathological features are summarized in Table 1. Among these 70 patients, 68.6% (48 patients) were < 65 years of age, 65.7% (46 patients) were male, and 62.9% (44 patients) had a smoking history. According to the World Health Organization classification criteria [23], 40 (57.1%) patients were diagnosed with adenocarcinoma, and 30 (42.9%) were diagnosed with non-adenocarcinoma. Regarding grade, 28 (40.0%) cases were well differentiated, 38 (54.3%) were poorly differentiated, and this information was missing in 4 (5.7%) cases. Based on the 7th edition of TNM staging system recommended by the International Association for the Study of Lung Cancer [24], 67.1% (47 patients) were stage I–II and 32.9% (23 patients) were stage III. There was no statistically significant difference in the demographic characteristics between the relapse-free group and brain metastatic group.

**Table 1:** Clinicopathologic features of 70 patients with non-small cell lung cancer

Characteristics	Case, n (%)	Relapse-free cases, n (%)	Brain metastasis cases, n (%)	*P* value
Age, years				0.848
< 65	48 (68.6)	23 (47.9)	25 (52.1)	
≥ 65	22 (31.4)	10 (45.5)	12 (54.5)	
Median (5th–95th)	60 (49.0–71.5)	60 (46.6–73.1)	60 (48.8–72.9)	0.693^a^
Sex				0.729
Male	46 (65.7)	21 (45.7)	25 (54.3)	
Female	24 (34.3)	12 (50.0)	12 (50.0)	
Smoking				0.713
Never	26 (37.1)	13 (50.0)	13 (50.0)	
Ever/current	44 (62.9)	20 (45.5)	24 (54.5)	
Histology				0.678
Adenocarcinoma	40 (57.1)	18 (45.0)	22 (55.0)	
Non-adenocarcinoma	30 (42.9)	15 (50.0)	15 (50.0)	
Grade				0.227
Well	28 (40.0)	16 (57.1)	12 (42.9)	
Poor	38 (54.3)	16 (42.1)	22 (57.9)	
Missing	4 (5.7)			
Clinical stage				0.667
I–II	47 (67.1)	23 (48.9)	24 (51.1)	
III	23 (32.9)	10 (43.5)	13 (56.5)	

^a^The *P* value was calculated using a Mann–Whitney U test. Other *P* values were calculated using a Chi square test

### Genes related to brain metastasis

**Fig. 1:**
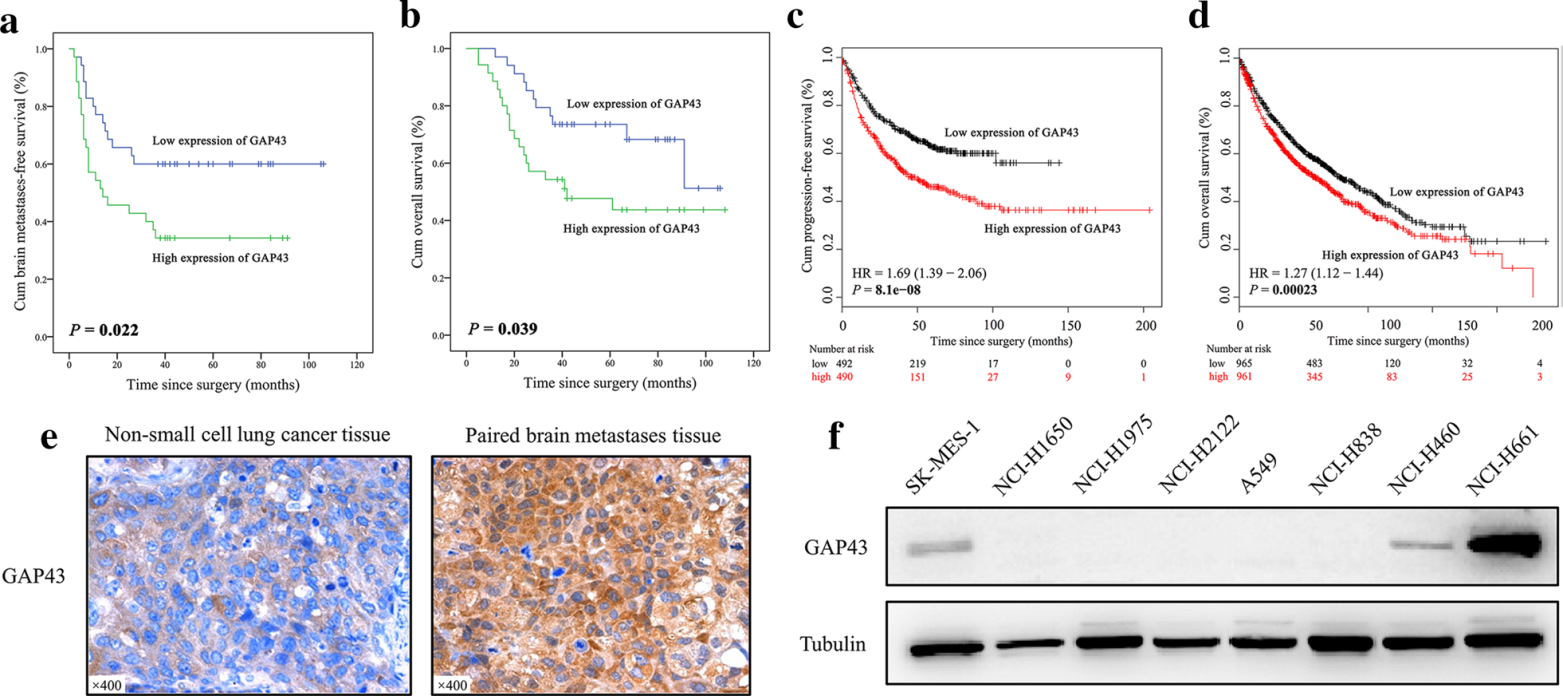
Expression of GAP43 in NSCLC. **a**, **b** Brain metastasis-free and overall survival curves of 70 patients with NSCLC based on the expression levels of GAP43. The *P* values were calculated using a log-rank test. **c**, **d** Progression-free and overall survival curves according to the expression levels of GAP43 in patients with lung cancer generated by a public database and web application called KM plotter (http://kmplot.com/analysis/index.php?p=service&cancer=lung). **e** Representative images of immunohistochemistry staining for GAP43 expression in primary NSCLC tissues and corresponding brain metastasis tissues; magnification, ×400. A total of 3/5 brain metastasis tissues showed an elevated level of GAP43 expression compared with paired NSCLC tissues. **f** Expression levels of GAP43 in NSCLC cells evaluated by western blotting

The mRNA expression levels of 36 candidate genes were measured with a Luminex assay, and a Cox regression model was applied to estimate the prognostic value of each gene. As presented in Table 2, univariate Cox regression analysis indicated that GAP43 and PMP2 were risk genes for NSCLC brain metastasis (crude HR: 2.12; 95% CI 1.09–4.13; *P* = 0.027 for GAP43; crude HR: 2.12; 95% CI 1.05–4.30; *P* = 0.036 for PMP2). Additionally, ST6GALNAC5 (*P* = 0.071), SNAI1 (*P* = 0.066), CDH2 (*P* = 0.073) and FABP7 (*P* = 0.066) showed borderline significant predictive values. Furthermore, multivariate Cox regression analysis involving genes whose *P* values were less than 0.1 in univariate analyses and clinicopathologic factors demonstrated that GAP43, SNAI1 and grade were independent prognostic factors for NSCLC brain metastasis (Table 2). The risk of developing brain metastases for NSCLC patients with high GAP43 expression levels was 3.29-fold higher than that for patients with low GAP43 levels (95% CI 1.55–7.00; *P* = 0.002). Compared with NSCLC patients with low SNAI1 expression levels, those with high expression levels had a 1.98-fold higher risk of developing brain metastases (95% CI 1.00–3.95; *P* = 0.051). Patients with poorly differentiated tumors more easily developed brain metastases than those with well-differentiated tumors (adjusted HR: 2.08; 95% CI 1.00–4.34; *P* = 0.051). Furthermore, Kaplan–Meier survival curves and the results from a public database (http://kmplot.com) indicated that patients with high GAP43 levels showed worse progression-free and overall survival (Fig. 1a–d).

**Table 2:** Univariate and multivariate Cox regression analyses of the risk genes for non-small cell lung cancer brain metastasis

Factors^a^	HR	95% CI	*P* value
Univariate analysis			
BMI1	0.58	0.29–1.16	0.124
POU5F1	0.75	0.38–1.47	0.397
KLF4	1.11	0.57–2.18	0.757
SMO	0.86	0.43–1.68	0.650
JAG1	0.85	0.43–1.67	0.634
SPARC	1.44	0.73–2.84	0.292
ST6GALNAC5	1.90	0.95–3.81	0.071
SNAI1	1.89	0.96–3.74	0.066
TNF	1.23	0.63–2.41	0.545
CXCR5	0.77	0.39–1.51	0.448
GFAP	1.00	0.51–1.96	0.995
DYNC1LI2	1.13	0.58–2.21	0.729
MMP3	1.21	0.62–2.37	0.579
CDH2	1.87	0.94–3.71	0.073
EGFR	0.65	0.33–1.28	0.212
VCAN	1.50	0.76–2.97	0.247
HGF	0.76	0.39–1.50	0.427
CTNNB1	0.76	0.39–1.50	0.428
CXCR4	1.46	0.74–2.88	0.275
KIF3A	1.47	0.74–2.89	0.269
GPM6A	0.70	0.36–1.38	0.307
HBEGF	0.95	0.48–1.86	0.869
CXCL13	0.99	0.51–1.95	0.985
TGFB2	0.92	0.47–1.80	0.803
PLP1	0.81	0.39–1.69	0.568
TWIST1	1.45	0.74–2.86	0.280
PTGS2	0.87	0.44–1.71	0.679
BPTF	0.77	0.39–1.52	0.443
FABP7	1.92	0.96–3.83	0.066
GAP43	2.12	1.09–4.13	*0.027*
PMP2	2.12	1.05–4.30	*0.036*
UGT8	1.23	0.63–2.41	0.549
GPM6B	1.00	0.51–1.96	0.990
FAM107A	0.62	0.31–1.23	0.169
IL8	1.27	0.65–2.49	0.492
MMP9	1.16	0.59–2.27	0.668
Multivariate analysis^b^			
GAP43	3.29	1.55–7.00	*0.002*
SNAI1	1.98	1.00–3.95	0.051
Grade	2.08	1.00–4.34	0.051

*HR* hazard ratio, *CI* confidence interval

Italic values were statistically significant (*P* < 0.05)

^a^Factors were analyzed as categorical variables, and low-expression groups or well-differentiated groups were referred

^b^Genes whose *P* values were < 0.1 in univariate analyses and clinicopathologic factors were involved in the multivariate Cox regression analysis, and the Forward LR (based on partial maximum likelihood estimation) was used to identify independent risk factors

### GAP43 expression in brain metastatic tissues and NSCLC cell lines

To further investigate the role of GAP43 in NSCLC, 5 FFPE NSCLC tissues with paired brain metastatic tissues and a panel of human NSCLC cell lines were collected. Immunohistochemistry showed that 3/5 brain metastatic tissues had a higher level of GAP43 expression than the corresponding primary NSCLC tissues (Fig. 1e). Additionally, as illustrated in Fig. 1f, GAP43 expression was high in NCI-H661 cells, comparatively lower in SK-MES-1 and NCI-H460 cells and absent in NCI-H1650, NCI-H1975, NCI-H2122, A549 and NCI-H838 cells.

### GAP43 promoted cell migration and invasion in vitro

**Fig. 2:**
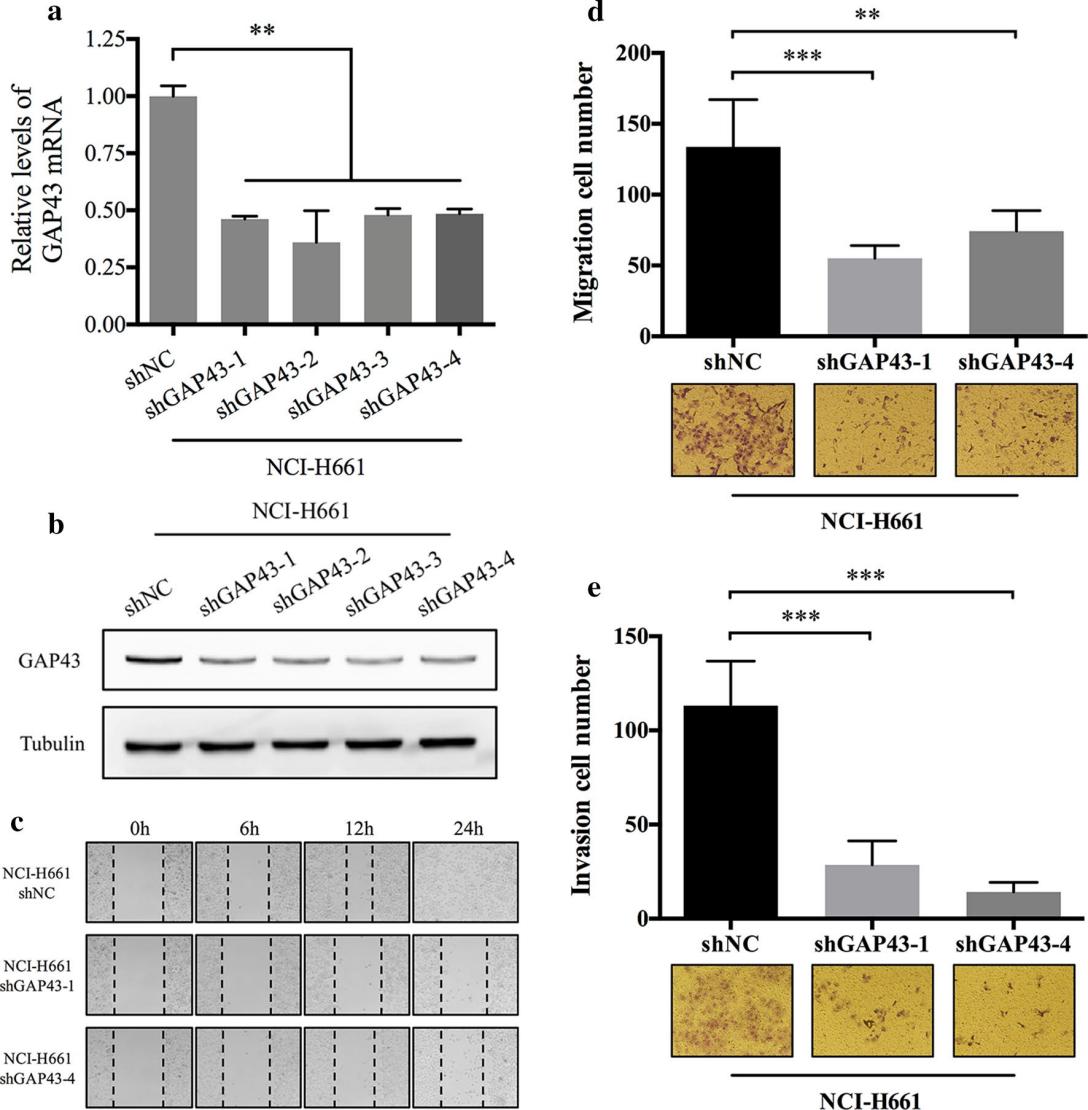
GAP43 knockdown suppressed cell migration and invasion. **a**, **b** Verification of transient GAP43 knockdown in NCI-H661 cells by qPCR and western blotting. **c**–**e** Wound healing and transwell migration and invasion assays showing that GAP43 knockdown suppresses NCI-H661 cell migration and invasion; representative photos are presented. All *P* values were estimated with Student’s t-test. **P < 0.01, ***P < 0.001

**Fig. 3:**
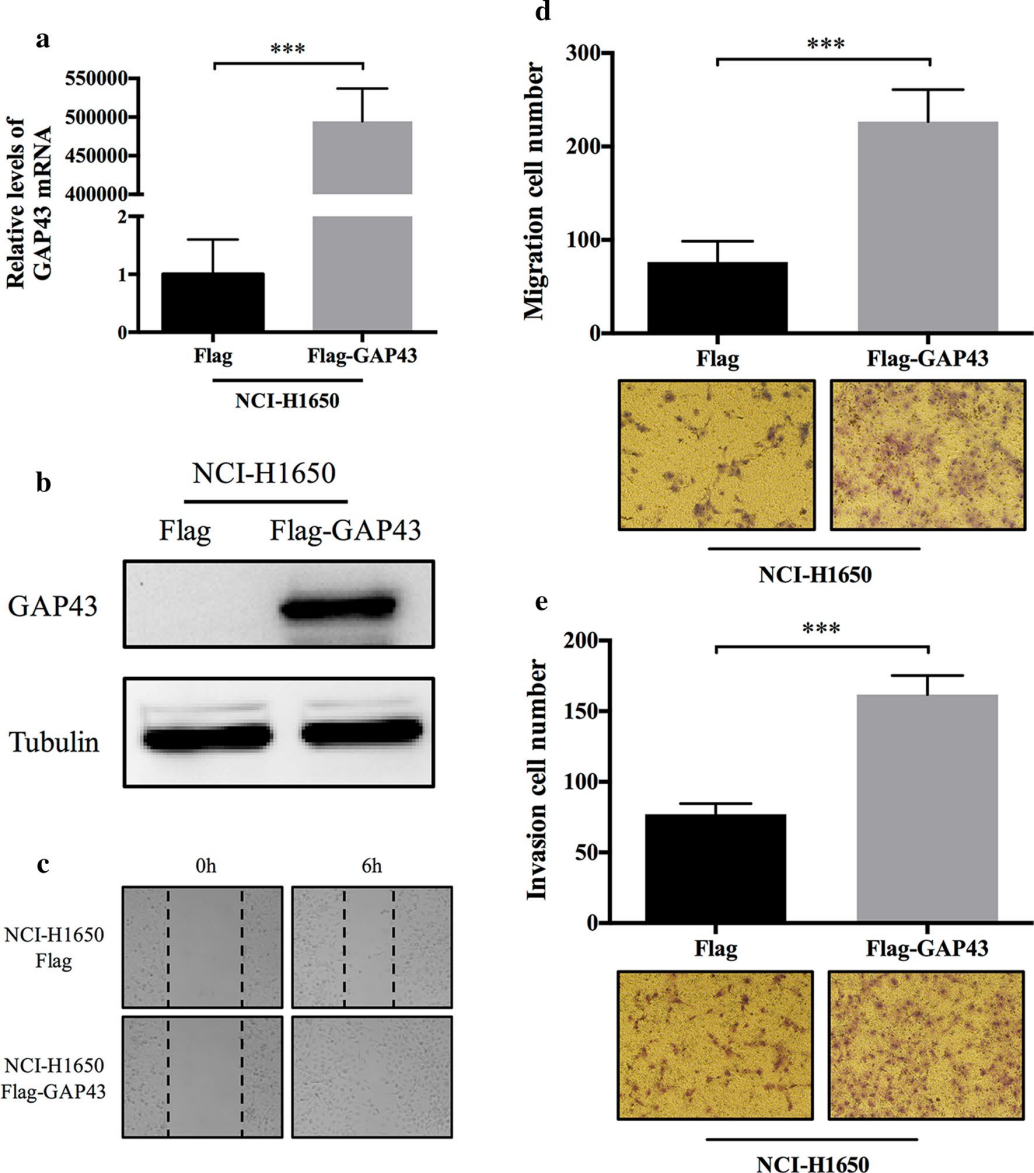
GAP43 overexpression enhanced cell migration and invasion abilities. **a**, **b** Confirmation of transient GAP43 overexpression in NCI-H1650 cells at the mRNA and protein levels. **c**–**e** Representative images of the wound healing and transwell migration and invasion assays demonstrate that GAP43 overexpression enhanced NCI-H1650 cell migration and invasion abilities. All *P* values were estimated with Student’s t-test. ***P < 0.001

To study the effect of GAP43 on cell migration and invasion in vitro, NCI-H661 and NCI-H1650 cells were chosen to perform transient GAP43 knockdown and overexpression, respectively. The suppression of GAP43 in NCI-H661 cells and overexpression of GAP43 in NCI-H1650 cells were confirmed at both the mRNA and protein levels (Figs. 2a, b, 3a, b). As revealed in a wound-healing assay (Fig. 2c), GAP43 knockdown in NCI-H661 cells significantly impaired cell lateral migration ability. Additionally, Transwell assays demonstrated that GAP43-silenced NCI-H661 cells migrated and invaded less efficiently than control cells (Fig. 2d, e). Similarly, GAP43 significantly heightened the migratory and invasive capacities of NCI-H1650 cells (Fig. 3c–e).

### GAP43 depletion inhibited metastasis in vivo

**Fig. 4:**
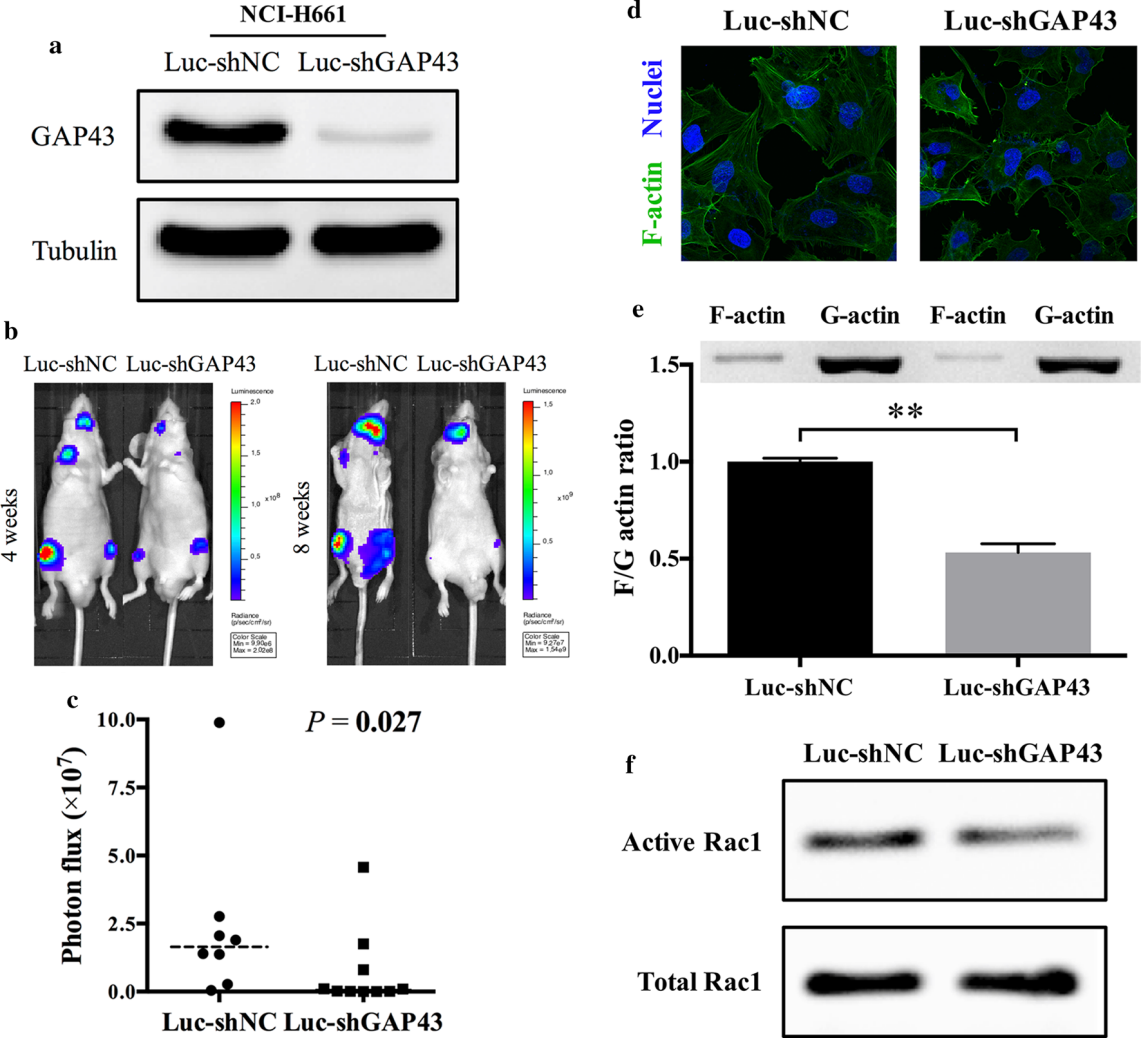
GAP43 depletion decreased metastasis in vivo and triggered F-actin depolymerization. **a** Validation of stable GAP43 knockdown in NCI-H661 cells by western blotting. **b** Representative images of bioluminescence 4 and 8 weeks after left ventricular injection of NCI-H661-Luc-shNC and NCI-H661-Luc-shGAP43 cells. **c** Quantification of bioluminescence in the 8th week. Each group had 10 mice, while 2 mice in the NCI-H661-Luc-shNC group died soon after the injection of left ventricle. The *P* value was determined using a Mann–Whitney U test. **d** Representative images of F-actin immunofluorescence in NCI-H661-Luc-shNC and NCI-H661-Luc-shGAP43 cells. **e** Expression levels of F-actin and G-actin in NCI-H661-Luc-shNC and NCI-H661-Luc-shGAP43 cells examined by western blotting and a histogram of the F-actin/G-actin ratio. F-actin was depolymerized and decreased after GAP43 knockdown. The *P* value was estimated with Student’s t-test. **f** Detection of active and total Rac1 in NCI-H661-Luc-shNC and NCI-H661-Luc-shGAP43 cells by western blotting. Active Rac1 decreased and total Rac1 remained unchanged after GAP43 depletion. **P < 0.01

To test and verify the function of GAP43 in vivo, NCI-H661 cells were stably transfected with luciferase-expressing GAP43 knockdown lentiviral vectors (Fig. 4a) and injected into the left ventricle of nude mice. Markedly, both brain and bone metastases decreased after GAP43 was silenced in NCI-H661 cells (Fig. 4b, c).

### GAP43 knockdown triggered F-actin depolymerization

To further explore the molecular mechanisms, we investigated epithelial-mesenchymal transition (EMT) markers, including E-cadherin, N-cadherin, fibronectin and vimentin, applied a Human Phospho-Kinase Array and Human XL Cytokine Antibody Array from R&D, Minneapolis, MN, USA. However, no significant difference was found related to tumor migration (data no shown).

To examine alterations in cytoskeletal organization, F-actin in stable GAP43-knockdown NCI-H661 cells was stained with phalloidin, and a marked structural change was observed due to decreased filamentous F-actin after GAP43 knockdown (Fig. 4d). Furthermore, an F-actin/G-actin ratio assay showed similar results, specifically, the amount of F-actin was reduced with GAP43 depletion (Fig. 4e). Next, a Rac1 activation assay was performed and showed that active Rac1 levels were decreased and total Rac1 stayed invariant after GAP43 was silenced (Fig. 4f).

## Discussion

Due to the high incidence and poor prognosis of brain metastasis, exploration of the underlying mechanisms and identification of potential predictive biomarkers and therapeutic targets is urgent. In our previous retrospective study, we examined 637 operable NSCLC patients and developed a nomogram to predict the occurrence of brain metastases and identified high-risk NSCLC patients for prophylactic cranial irradiation treatment [17]. To further extend these findings, the present study investigated the transcriptional profiles of 70 patients using a high-throughput Luminex assay and identified two genes, GAP43 and SNAI1, as independent predictors of NSCLC brain metastasis.

SNAI1 is a zinc-finger transcription factor that is important in EMT [25] because it transcriptionally restrains the adherent junction protein E-cadherin and elevates the levels of mesenchymal markers, such as vimentin and MMPs [25–28]. SNAI1 can induce tumorigenesis, promote invasion and migration, facilitate tumor development and impair sensitivity to chemotherapeutic drugs [29–31], and high SNAI1 expression was shown to be correlated with poor outcomes in diverse tumor types [29, 32, 33]. Similar to the present research, Jeevan et al. [34] found that SNAI1 expression is elevated in brain metastatic tissues compared with that in non-metastatic lung tissues.

GAP43 is an axonal membrane protein expressed in neuronal growth cones that plays a vital role in neural growth, axonal regeneration and stabilization of synaptic function [35, 36]. Given its specific expression in neurons, GAP43 is often used to differentiate between nerve sheath and non-nerve sheath neoplasms in the brain [37], and it also serves to detect minimal residual disease in neuroblastoma [38]. However, few studies have examined the prognostic value of GAP43 in tumors. The current study investigated the transcriptional levels of GAP43 in NSCLC tissues and found that GAP43 was significantly overexpressed in tissues that showed a tendency toward brain metastasis, and Kaplan–Meier analyses showed that GAP43 is also related to overall survival. These results were further confirmed by a public data set. Consistent with our study, Klein et al. [18] applied the gene expression profiles of 8 bone and 18 brain metastatic lesions from primary breast cancer and found that GAP43 was expressed in brain metastases but not in bone metastases, concluding that GAP43 might be a brain-specific metastasis gene.

The present study showed that both lateral and transmembrane cell migration was facilitated by GAP43 in vitro, suggesting positive effects of GAP43 on cell motility. Further, to test and validate the role of GAP43 in vivo, intracarotid injection was performed on mice initially but exhibited high mortality due to technological limitations. Therefore, instead, left ventricular injection was used to construct an in vivo brain metastasis model. As revealed by the BLI, cells colonized the brain and bone after injection, a finding that is in accordance with the metastatic patterns of lung cancer [39]. After 8 weeks of growth, both the brain metastatic and bone metastatic lesions in the GAP43-silenced group were smaller than those in the control group. This phenomenon indicated that knockdown of GAP43 could suppress the development of metastases.

To explore the underlying mechanisms, we first investigated markers of EMT, which is currently well acknowledged to be associated with the onset of cancer cell migration [40]. However, NSCLC cells did not exhibit an altered molecular signature after GAP43 knockdown or overexpression, suggesting that EMT may not be involved in the migration promoted by GAP43. Additionally, cells often migrate in response to specific chemical signals, and it has been reported that brain metastatic cancer cells can secrete certain cytokines to break the tight junctions of the BBB to facilitate colonization in the brain [12, 15, 41]. To probe significant molecules, Human XL Cytokine Antibody Arrays were utilized to examine NSCLC cell medium. However, the cytokine content in the medium did not change after GAP43 expression was artificially altered, hinting that GAP43 cannot trigger cytokine release. To identify other possible pathways, we employed Human Phospho-Kinase Arrays to assess protein content in NSCLC cells, but no significant molecular changes were found to be related to tumor migration (data not shown).

Next, we wondered whether GAP43 could regulate cytoskeletal organization to promote migration. Cellular actin exists in filamentous (F-actin) and globular (G-actin) forms, and the filamentous form is the major component of the actin cytoskeleton. G-actin polymerizes to form F-actin, and the F-actin/G-actin ratio regulates various functions, including cell motility [42]. To validate the notion that GAP43 regulates cytoskeletal organization, the F-actin cytoskeleton was stained, and an F-actin/G-actin ratio assay was performed, both of which showed that F-actin depolymerized and decreased after GAP43 was knocked down. These phenomena suggested that GAP43 may promote cell migration by preventing F-actin cytoskeleton depolymerization. According to the literature, during the course of axonal pathfinding, GAP43 directly binds to F-actin, thereby preventing filament depolymerization and facilitating pseudopodium formation [43, 44], which also confirms our findings. In addition, the Rho GTPase family has a well-recognized role in regulation of cellular motility by controlling actin dynamics [45]. Rho GTPase activation assays indicated that activated Rac1 was decreased after GAP43 was silenced. As reported, active Rac1 can interact with the WAVE regulatory complex, leading to activation of the actin nucleation complex Arp2/3 and thus actin assembly [46]. Therefore, it is speculated that GAP43 prevents F-actin depolymerization by activating Rac1, thereby promoting migration.

## Conclusions

The present study demonstrates that GAP43 is a brain metastasis-related gene and a potential therapeutic target in NSCLC and that GAP43 may contribute to metastasis by controlling F-actin dynamics via Rac1 activation. Further detailed mechanisms still need to be elucidated.

## Abbreviations

NSCLC: non-small cell lung cancer
GAP43: growth-associated protein 43
BBB: blood–brain barrier
MMP: matrix metalloproteinase
PLGF: placental growth factor
VEGF: vascular endothelial growth factor
FFPE: formalin-fixed and paraffin-embedded
STR: short tandem repeat
PBS: phosphate-buffered saline
RT-qPCR: reverse transcription-quantitative polymerase chain reaction
BLI: bioluminescence imaging
EMT: epithelial–mesenchymal transition
